# ERK promotes miR34a/ISOC1 signaling to enhance all trans retinoic acid-induced myeloid differentiation in acute promyelocytic leukemia

**DOI:** 10.1016/j.jbc.2026.113284

**Published:** 2026-06-20

**Authors:** Yu Zhang, Yaxin Li, Na Li, Cunru Zou, Chengyue Liu, Xiaoyun Zhang, Xiaodi Ding, Rongxuan Cao, Liping Liu, Xuehong Ran, Zhenzhen Lin, Wenxia Su

**Affiliations:** 1 Department of Physiology, School of Basic Medicine, Shandong Second Medical University, Weifang, China; 2 Department of Clinical Laboratory, Weifang Central Blood Station, Weifang, China; 3 Department of Clinical Laboratory, Weifang Maternal and Child Health Hospital, Weifang, China; 4 Department of Pathology, School of Basic Medicine, Shandong Second Medical University, Weifang, China; 5 Department of Hematology, Weifang People’s Hospital, Weifang, China; 6 Key Laboratory of Immune Microenvironment and Inflammatory Disease Research in Universities of Shandong Province, School of Basic Medicine, Shandong Second Medical University, Weifang, China; 7 Functional Science Laboratory, School of Basic Medicine, Shandong Second Medical University, Weifang, China

**Keywords:** acute promyelocytic leukemia, all trans retinoic acid, CDK6, ERK, ISOC1, miR34a

## Abstract

While all-trans retinoic acid (ATRA) is successfully used to treat acute promyelocytic leukemia (APL), therapeutic resistance remains a major clinical challenge, highlighting an urgent need to identify the regulators of ATRA efficiency. In the present study, we analyzed paired bone marrow samples of patients with APL and found that miR34a isoforms (-3p and -5p) were significantly elevated at complete remission compared to initial diagnosis. Overexpression of pre-miR34a enhanced ATRA-induced myeloid differentiation in APL cells as well as in xenograft models. Mechanistically, we found that miR34a promoted G1/S cell cycle arrest and reduced the protein level of CDK6, a validated target of miR34a-5p. Furthermore, bioinformatic analysis predicted ISOC1 was a target of miR34a-3p, and dual-luciferase reporter assays validated the direct binding of miR34a-3p to the 3′ UTR of *ISOC1*. Gene set enrichment analysis (GSEA) illustrated "hematopoietic cell lineage" pathway was significantly enriched in patients with acute myeloid leukemia (AML) having low levels of *ISOC1*. Knockdown of *ISOC1* enhanced ATRA-induced myeloid differentiation, and rescue experiments confirmed that miR34a regulated ATRA efficiency by targeting ISOC1. Moreover, ERK inhibition blocked the ability of miR34a to enhance ATRA-induced myeloid differentiation. Consistently, ERK inactivation significantly reduced miR34a-3p levels while increased ISOC1 levels in the presence of ATRA. Taken together, Our findings demonstrated that miR34a enhanced ATRA-induced myeloid differentiation by targeting CDK6 *via* miR34a-5p and ISOC1 *via* miR34a-3p, ERK signaling promoted ATRA-induced myeloid differentiation through miR34a-3p/ISOC1 axis. Targeting this regulatory axis may provide a novel combinatorial strategy to improve the therapeutic efficacy of ATRA in APL.

Acute promyelocytic leukemia, characterized by the PML-RARα oncoprotein, has been transformed into a highly curable malignancy through differentiation therapy with ATRA (1). The PML-RARα oncoprotein induces a differentiation blockade at the promyelocytic stage by recruiting transcriptional repressor complexes, thereby disrupting physiological retinoic acid signaling (2). ATRA binds to PML-RARα, converts PML-RARα from a transcriptional repressor to a transcriptional activator, and induces subsequent degradation of PML-RARα, reactivating the expression of key differentiation drivers, ultimately inducing terminal granulocytic differentiation (3, 4). Despite its remarkable efficacy, approximately 10 to 20% of patients develop resistance to ATRA. However, the regulators modulating ATRA efficiency remain incompletely understood (5, 6).

MicroRNAs (miRNAs) are evolutionarily highly conserved small single-stranded ncRNAs of ∼ 22 nt in length, repressing target mRNA translation or promoting target mRNA decay (7). They are critical modulators of hematopoietic differentiation (8, 9) and also important regulators of ATRA efficiency (10, 11). MiR34a, a well-characterized tumor suppressor frequently downregulated in diverse malignancies such as neuroblastoma (12), breast cancer (13), and ovarian cancer (14), is known to target more than 30 proteins, including CDK6, MYC, MET, NOTCH1, and BCL2, to regulate cell cycle, cell differentiation, and cell apoptosis (15, 16, 17). However, the role and mechanism of miR34a in regulating ATRA efficiency are still elusive.

ERK signaling, a central pathway in eukaryotic cells that governs proliferation, differentiation, and survival, is activated by retinoic acid (RA), and this activation is required for RA-induced cell differentiation (18). Conversely, ERK pathway also enhances the efficacy of RA (18, 19, 20). Besides, ERK also acts as a downstream effector of miR34a, mediating the proliferation of mesangial and K562 cells (21, 22). However, whether ERK signaling and miR34a interact to modulate the efficiency of ATRA remains unclear.

In this study, we performed clinical sample analysis and functional studies *in vitro* and *in vivo*. We found that miR34a isoforms (−3p and −5p) were significantly elevated in bone marrow samples from APL patients at complete remission compared to initial diagnosis. Overexpression of pre-miR34a enhanced ATRA-induced myeloid differentiation in APL cells and in xenograft models. Mechanistically, miR34a-5p promoted G1/S cell cycle arrest by targeting CDK6. In addition, using dual-luciferase reporter assay and rescue experiment, we identified miR34a-3p regulated ATRA efficiency by targeting ISOC1. Importantly, ERK inhibition blocked miR34a-3p/ISOC1 signaling and reduced the ability of miR34a to enhance ATRA-induced differentiation. These findings demonstrated that miR34a enhanced ATRA-induced myeloid differentiation by targeting CDK6 *via* miR34a-5p and ISOC1 *via* miR34a-3p, ERK signaling promoted ATRA-induced myeloid differentiation through miR34a-3p/ISOC1 axis.

## Results

### miR34a enhanced ATRA-induced myeloid differentiation in APL cells

**Figure 1:**
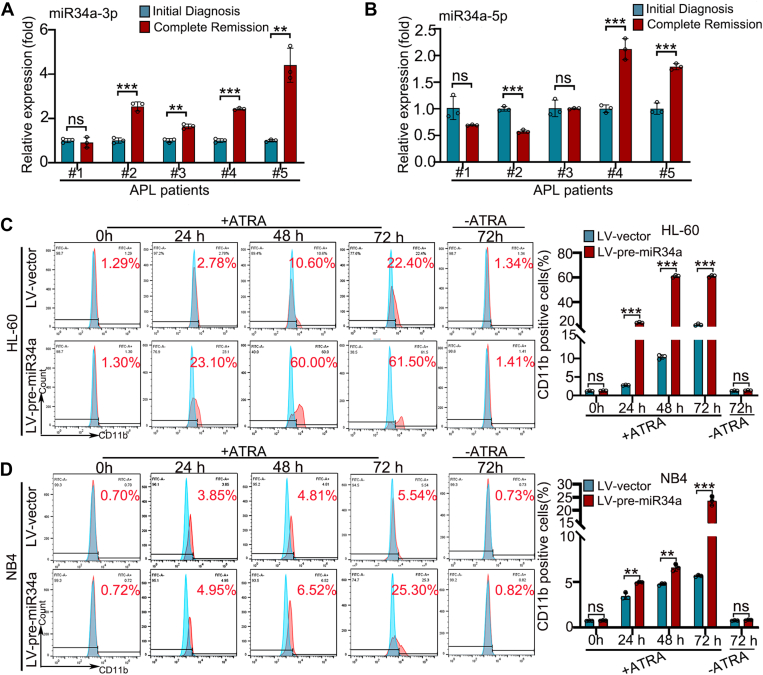
**miR34a enhanced ATRA-induced myeloid differentiation in APL cells.***A* and *B*, QRT-PCR analysis of miR34a-3p (*A*) and miR34a-5p (*B*) expression in paired bone marrow samples of five acute promyelocytic leukemia (APL) patients at two timepoints: initial diagnosis and complete remission. Unpaired *t* test was performed, data were shown as mean ± SD (n = 3 technical replicates). ns = not significant, ∗∗*p* < 0.01, ∗∗∗*p* < 0.001. *C* and *D*, flow cytometry analysis of CD11b expression (indicating myeloid differentiation) of HL-60 (*C*) and NB4 cells (*D*) stably infected with LV-pre-miR34a or LV-vector (control) after 2 μM ATRA treatment for 0 h, 24 h, 48 h, 72 h, or DMSO treatment for 72 h. Unpaired *t* test was performed, data were shown as mean ± SD (n = 3 biological replicates). ns = not significant, ∗∗*p* < 0.01, ∗∗∗*p* < 0.001.

Quantitative Real-Time PCR (QRT-PCR) analysis of miR34a-3p and miR34a-5p in paired bone marrow samples of 5 APL patients showed they were significantly higher at complete remission than at initial diagnosis. An increase in mRNA levels at complete remission was observed for miR34a-3p in four patients (Fig. 1*A*) and for miR34a-5p in two patients (Fig. 1*B*). We then investigated the impact of miR34a on the efficiency of ATRA in APL by establishing stable miR34a-overexpressing APL cells through lentiviral infection of HL-60 and NB4 cells with LV-pre-miR34a. Cells stably infected with LV-vector were used as controls. In HL-60 cells, the percentage of CD11b^+^ cells showed no difference between controls and miR34a-overexpressing cells without ATRA treatment. However, in the presence of ATRA, CD11b^+^ cells increased from 2.79 ± 0.11% (control) to 23.3 ± 0.44% (LV-pre-miR34a) at 24 h (*p* < 0.001), from 10.42 ± 0.59% to 61.03 ± 0.93% at 48 h (*p* < 0.001), and from 21.7 ± 0.72% to 61.13 ± 0.81% at 72 h (*p* < 0.001) (Fig. 1*C*). Similarly, there is no differentiation difference between controls and miR34a-overexpressing NB4 cells without ATRA treatment. However, in the presence of ATRA, CD11b^+^ cells increased from 3.43 ± 0.37% (control) to 4.92 ± 0.10% (LV-pre-miR34a) at 24 h (*p* < 0.01), from 4.73 ± 0.13% to 6.55 ± 0.39% at 48 h (*p* < 0.01), and from 5.64 ± 0.10% to 23.53 ± 1.75% at 72 h (*p* < 0.001) (Fig. 1*D*). Together, these results suggested that enforced expression of miR34a significantly enhanced ATRA-induced myeloid differentiation in APL cells.

### miR34a enhanced ATRA-induced myeloid differentiation in NOG xenograft mouse models

**Figure 2:**
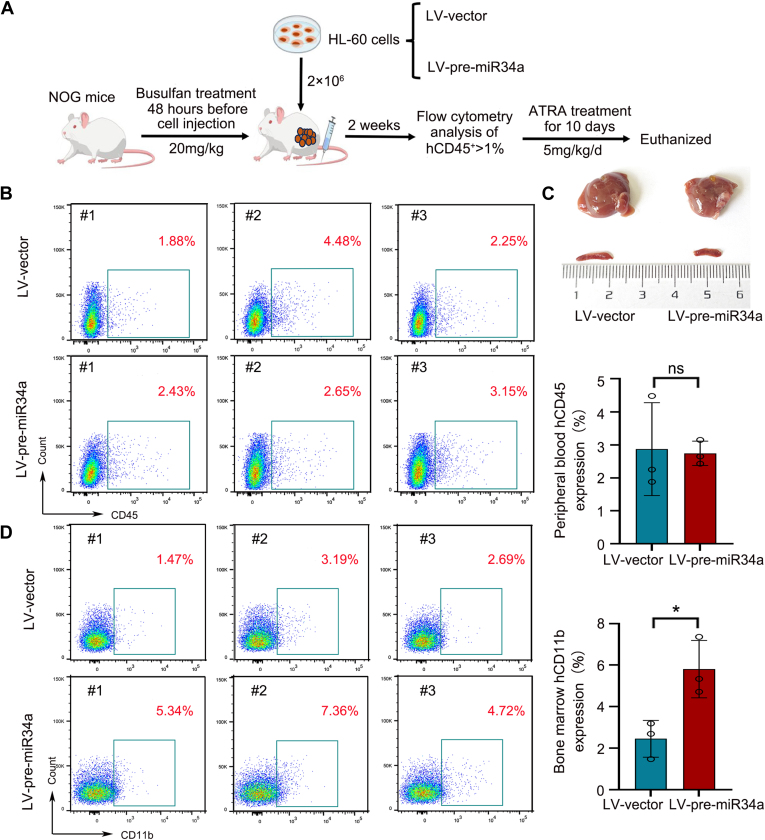
**miR34a enhanced ATRA-induced myeloid differentiation in NOG xenograft mouse models.***A*, schematic diagram of NOG mouse xenograft model establishment and ATRA administration. *B*, flow cytometry analysis of peripheral blood human CD45^+^ (hCD45^+^) cell percentages in LV-vector and LV-pre-miR34a groups 2 weeks after transplantation. Flow cytometry plots for individual mice (#1, #2, #3) were shown. Unpaired *t* test was performed, data were shown as mean ± SD (n = 3 biological replicates). ns = not significant. *C*, representative images of liver and spleen from LV-vector and LV-pre-miR34a group mice after 10 days of ATRA treatment. *D*, flow cytometry analysis of human CD11b (hCD11b) expression in bone marrow from recipient mice after ATRA treatment. Unpaired *t* test was performed, data were shown as mean ± SD (n = 3 biological replicates). ∗, *p* < 0.05.

To examine the effect of miR34a on myeloid differentiation of APL cells *in vivo*, NOD.Cg-Prkdc^scid^Il2rg^tm1Sug^/JicCrl (NOG) mice (6 weeks old, body weight 16–18*g*) were transplanted with HL-60 cells stably infected with either LV-vector or LV-pre-miR34a lentivirus. Two days prior to tail vein injection, mice were treated with busulfan (20 mg/kg) to enhance cell engraftment (23). Two weeks after transplantation, peripheral blood human CD45 (hCD45) levels were analyzed by flow cytometry. Once hCD45^+^ cells exceeded 1%, mice were treated with ATRA (5 mg/kg/d) for 10 days to induce myeloid differentiation (24) (Fig. 2*A*). The results showed that hCD45^+^ cells accounted for 2.87 ± 1.41% in the LV-vector group and 2.74 ± 0.37% in the LV-pre-miR34a group 2 weeks after transplantation, indicating comparable engraftment efficiency between the two groups (*p* > 0.05) (Fig. 2*B*). After 10 days of ATRA treatment, both liver and spleen size were reduced in the LV-pre-miR34a group compared with the LV-vector group (Fig. 2*C*). Furthermore, flow cytometry analysis of bone marrow mononuclear cells (BMMNCs) revealed that the percentage of human CD11b^+^ cells was significantly higher in the LV-pre-miR34a group (5.81% ± 1.38%) than in the LV-vector group (2.45% ± 0.88%), with *p* < 0.05 (Fig. 2*D*). Collectively, these results demonstrated that miR34a enhanced ATRA-induced differentiation of APL cells *in vivo*.

### miR34a induced G1/S cell cycle arrest *via* targeting CDK6

**Figure 3:**
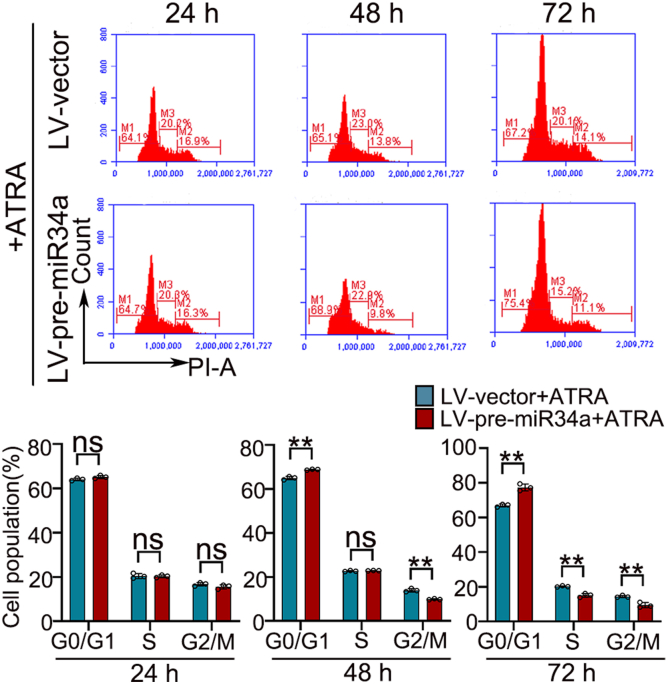
**miR34a induced G1/S cell cycle arrest in APL cells with ATRA treatment.** Cell cycle was analyzed by flow cytometry in HL-60 cells stably infected with LV-pre-miR34a or LV-vector (control) after 2 μM ATRA treatment for 24 h, 48 h, and 72 h. M1: G0/G1 populations, M2: G2/M populations, M3: S populations. Unpaired *t* test was performed, data were shown as mean ± SD (n = 3 biological replicates). ns = not significant, ∗∗*p* < 0.01.

Given the established relationship between cell differentiation and cell cycle regulation, we investigated the impact of miR34a overexpression on cell cycle in APL cells treated with ATRA. Flow cytometry analysis revealed a significant accumulation of G0/G1 population in miR34a-overexpressing HL-60 cells at both 48 h (68.83 ± 0.21% in miR34a-overexpressing cells vs 65.00 ± 0.75% in controls, *p* < 0.01) and 72 h (77.30 ± 2.01% in miR34a-overexpressing cells vs 66.93 ± 0.83% in controls, *p* < 0.01). Meanwhile, a significant reduction of G2/M population was observed in miR34a-overexpressing HL-60 cells at both 48 h (9.90 ± 0.26% in miR34a-overexpressing cells vs 13.97 ± 0.76% in controls, *p* < 0.01) and 72 h (9.43 ± 1.47% in miR34a-overexpressing cells vs 14.40 ± 0.61% in controls, *p* < 0.01). The population was also decreased in miR34a-overexpressing HL-60 cells at 72 h (15.07 ± 1.11% in miR34a-overexpressing cells vs 20.20 ± 0.36% in controls, *p* < 0.01) (Fig. 3). Together, these results suggested that enforced expression of miR34a induced G1/S cell cycle arrest in APL cells.

**Figure 4:**
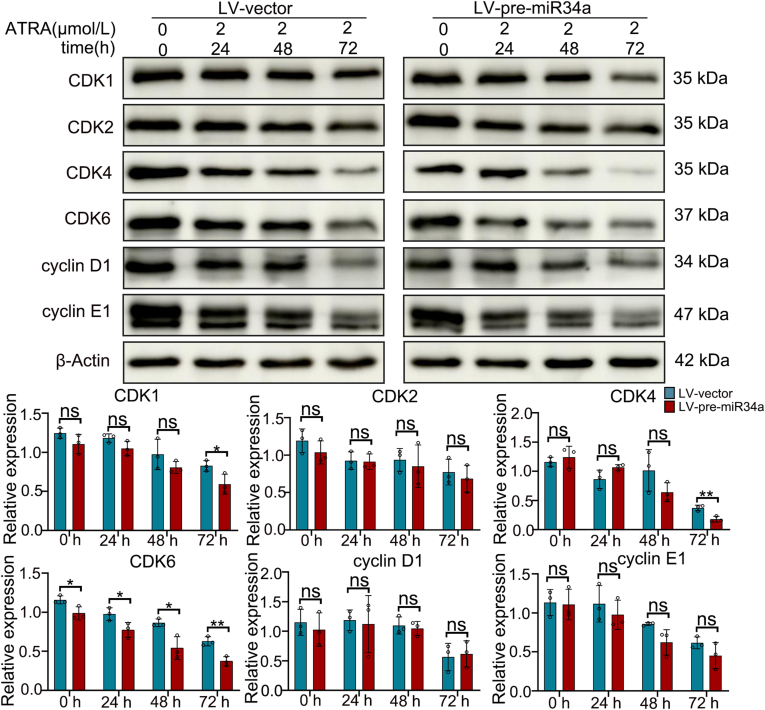
**miR34a inhibited the protein levels of CDK6 in APL cells.** HL-60 cells stably infected with LV-pre-miR34a or LV-vector (control) were not treated with ATRA (0 μM, 0 h) or treated with 2 μM ATRA for 24 h, 48 h, and 72 h. The protein levels of cell cycle regulators, including CDK1, CDK2, CDK4, CDK6, cyclin D1, and cyclin E1, were determined by Western blot. β-actin was used as a loading control. The relative protein levels (target protein/β-actin) of three biological replicates were shown by bar chart. Unpaired *t* test was performed, data were shown as mean ± SD (n = 3 biological replicates). ns = not significant, ∗*p* < 0.05 ∗∗*p* < 0.01.

Western blot analysis of cell cycle regulators in HL-60 cells showed no significant changes in protein levels of CDK2, cyclin D1, and cyclin E1 upon overexpression of miR34a, regardless of ATRA treatment. Compared to controls, reductions in CDK1 and CDK4 protein levels were observed in miR34a -overexpressing cells treated with ATRA for 72 h. Notably, ectopic expression of miR34a led to a significant decrease in CDK6, a validated miR34a-5p target (17), either with or without ATRA treatment (Fig. 4). Together, these results suggested that miR34a induced G1/S cell cycle arrest *via* targeting CDK6.

### ISOC1 was a novel target of miR34a-3p

**Figure 5:**
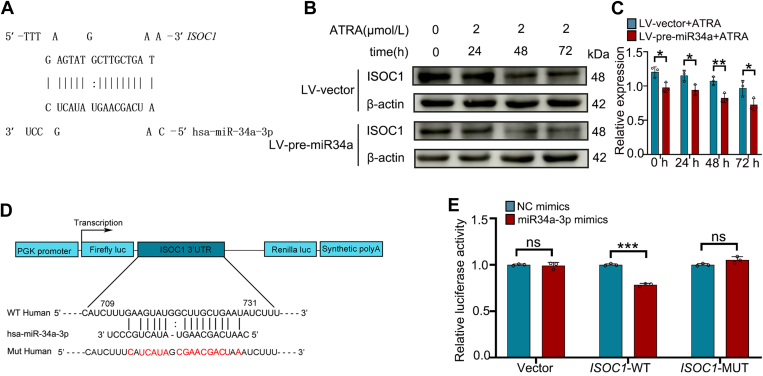
**ISOC1 was the target of miR34a-3p.***A*, prediction of a conserved binding site for miR34a-3p in the 3′ UTR of *ISOC1* in the miRanda database. *B*, western blot analysis of ISOC1 protein expression in miR34a-overexpressing HL-60 cells and controls, either untreated (0 μM ATRA, 0 h) or treated with 2 μM ATRA for 24, 48, or 72 h. β-actin was used as a loading control. *C*, relative protein levels (ISOC1/β-actin) of three biological replicates were shown by bar chart. Unpaired *t* test was performed, data were shown as mean ± SD (n = 3 biological replicates). ∗*p* < 0.05 ∗∗*p* < 0.01. *D*, schematic of the wild-type (WT) and mutant (MUT) *ISOC1* 3′ UTR luciferase reporter constructs used in the dual-luciferase reporter assay. *E*, results of the dual-luciferase reporter assay. Unpaired *t* test was performed, data were shown as mean ± SD (n = 3 biological replicates). ns = not significant, ∗∗∗*p* < 0.001.

Bioinformatic analysis using miRanda database predicted a conserved binding site for miR34a-3p in the 3′ UTR of *ISOC1* (Fig. 5*A*). The protein level of ISOC1 was markedly reduced in miR34a-overexpressing HL-60 cells either with or without ATRA treatment (Fig. 5, *B* and *C*). Dual-luciferase reporter assays demonstrated significant repression of luciferase activity (1.50-fold reduction, *p* < 0.001) when the *ISOC1* 3′ UTR was co-infected with miR34a-3p mimics compared to controls (Fig. 5, *D* and *E*). Together, these results suggested that ISOC1 was a target of miR34a-3p.

### ISOC1 was an inhibitor of ATRA-induced myeloid differentiation in APL cells

**Figure 6:**
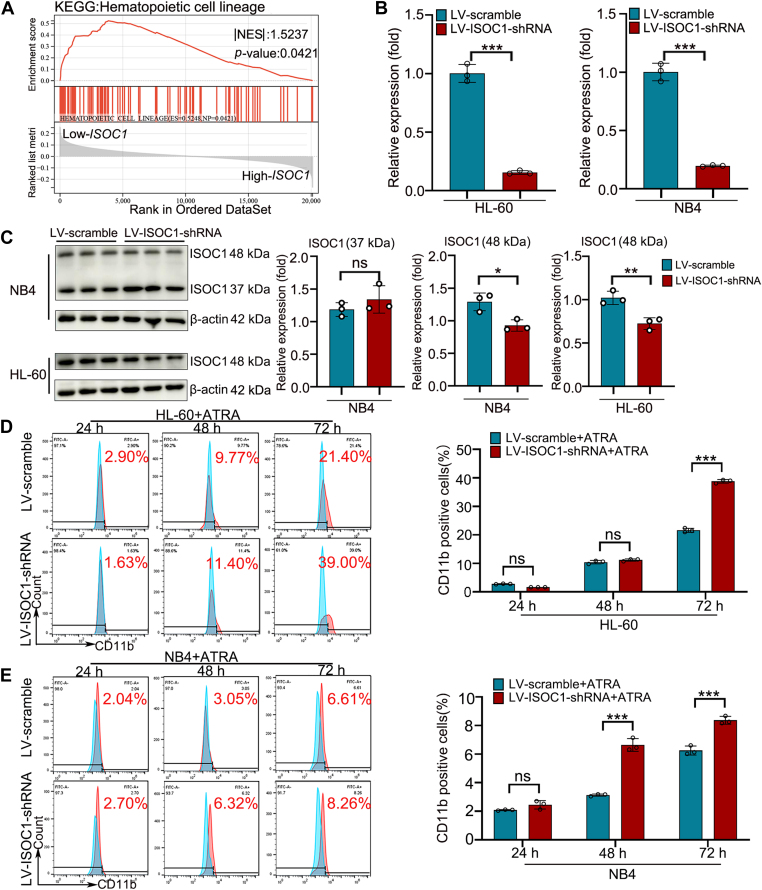
**ISOC1 was an inhibitor of ATRA-induced myeloid differentiation in APL cells.***A*, Gene Set Enrichment Analysis (GSEA) of AML samples from the TCGA database. *B*, HL-60 and NB4 cells were stably infected with LV-ISOC1-shRNA or LV-scramble (control). The knockdown efficiency was validated by QRT-PCR. Unpaired *t* test was performed, data were shown as mean ± SD (n = 3 technical replicates). ∗∗∗*p* < 0.001. *C*, knockdown efficiency of *ISOC1* was also validated by western blot. The relative protein levels (ISOC1/β-actin) of three biological replicates were shown by bar chart. Unpaired *t* test was performed, data were shown as mean ± SD (n = 3 biological replicates). ns = not significant, ∗*p* < 0.05 ∗∗*p* < 0.01. *D* and *E*, flow cytometry analysis of CD11b expression (indicating myeloid differentiation) in HL-60 cells (*D*) and NB4 cells (*E*) stably infected with LV-ISOC1-shRNA or LV-scramble (control) after 2 μM ATRA treatment for 24 h, 48 h, and 72 h. Unpaired *t* test was performed, data were shown as mean ± SD (n = 3 biological replicates). ns = not significant, ∗∗∗*p* < 0.001.

To elucidate the role of ISOC1 in ATRA-induced myeloid differentiation, we first performed gene set enrichment analysis (GSEA) using transcriptomic data from patients with AML. Among 186 KEGG pathways analyzed, the "hematopoietic cell lineage" pathway was significantly enriched in patients with low levels of *ISOC1* (|NES| = 1.5237, *p* < 0.05) (Fig. 6*A*), suggesting an inhibitory role of ISOC1 in hematopoietic differentiation.

To validate the function of ISOC1 in myeloid differentiation, we established stable *ISOC1*-silencing APL models through lentiviral infection of HL-60 and NB4 cells with LV-ISOC1-shRNA. Cells stably infected with LV-scramble were used as controls. Knockdown of *ISOC1* in both HL-60 and NB4 cells significantly reduced *ISOC1* mRNA levels (Fig. 6*B*). Western blot analysis showed that ISOC1 was detected as bands at both 37 kDa and 48 kDa. In NB4 cells, knockdown of *ISOC1* did not alter the 37 kDa band, while the 48 kDa band was markedly reduced. Similarly, in HL-60 cells, the 48 kDa band was also significantly decreased following *ISOC1* knockdown (Fig. 6*C*). Knockdown of *ISOC1* significantly enhanced ATRA-induced myeloid differentiation by 1.80-fold in HL-60 cells at 72 h (*p* < 0.001) and 1.34-fold in NB4 cells at 48 h (*p* < 0.001) compared to respective controls (Fig. 6, *D* and *E*). Together, these results suggested that ISOC1 was a critical negative regulator of ATRA-mediated myeloid differentiation in APL.

### miR34a enhanced ATRA-induced myeloid differentiation *via* targeting ISOC1

**Figure 7:**
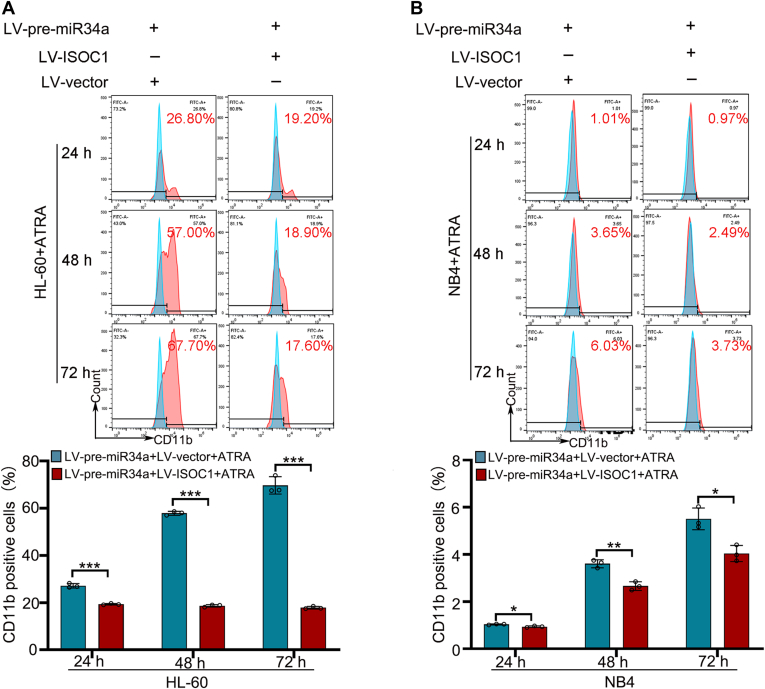
**miR34a enhanced ATRA-induced myeloid differentiation *via* targeting ISOC1 in APL cells.***A* and *B*, rescue experiment in HL-60 (*A*) and NB4 (*B*) cells. To confirm the role of ISOC1 in mediating the effect of miR34a on ATRA-induced myeloid differentiation, rescue experiments were conducted. Cells were co-infected with LV-pre-miR34a and either LV-ISOC1 (to overexpress ISOC1) or LV-vector control. Myeloid differentiation was assessed by flow cytometry analysis of CD11b expression following 2 μM ATRA treatment for 24 h, 48 h, and 72 h. Unpaired *t* test was performed, data were shown as mean ± SD (n = 3 biological replicates). ∗*p* < 0.05, ∗∗*p* < 0.01, ∗∗∗*p* < 0.001.

To functionally validate ISOC1 as a critical downstream effector of miR34a in enhancing ATRA-induced myeloid differentiation, we conducted rescue experiments in HL-60 and NB4 cells. Overexpression of miR34a alone promoted ATRA-induced differentiation in a time-dependent manner. However, concurrent expression of ISOC1 significantly reversed this effect. In HL-60 cells, ISOC1 co-expression reduced CD11b^+^ cells by 1.4-fold at 24 h, 3.1-fold at 48 h, and 3.9-fold at 72 h compared with miR34a alone (Fig. 7*A*). In NB4 cells, ISOC1 co-expression decreased differentiation rates by 1.1-fold at 24 h, and 1.4-fold at both 48 h and 72 h (Fig. 7*B*). Together, these results suggested that miR34a enhanced ATRA-induced myeloid differentiation *via* targeting ISOC1.

### Inhibition of ERK suppressed miR34a-3p/ISOC1 signaling to block ATRA-induced myeloid differentiation in APL cells

**Figure 8:**
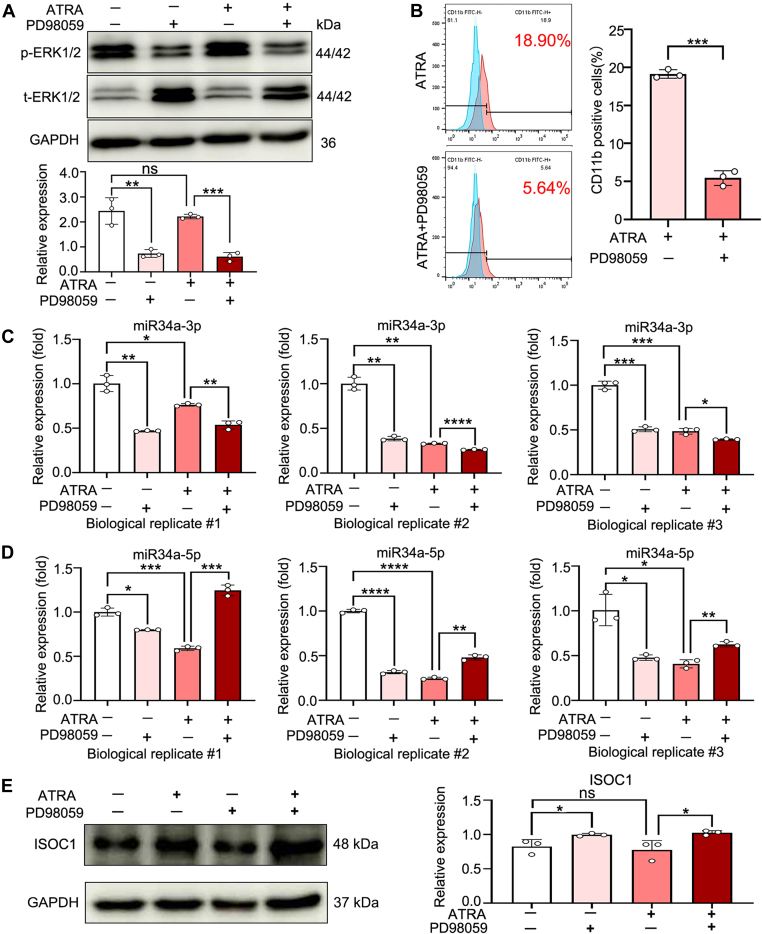
**Inhibition of ERK signaling suppressed miR34a expression to attenuate ATRA-induced myeloid differentiation in APL cells.***A*, western blot analysis of phosphorylated ERK (p-ERK) levels in HL-60 cells treated with 2 μM PD98059 (a MEK inhibitor) alone, or 1 μM ATRA alone, or a combination of 2 μM PD98059 and 1 μM ATRA for 48 h. HL-60 cells treated with DMSO were used as controls. The relative protein levels (p-ERK/t-ERK) of three biological replicates were shown in a bar chart. One-way analysis of variance (ANOVA) revealed a statistically significant difference among the four groups (F = 32.35, *p* < 0.0001), and an unpaired *t* test was performed between two groups; the data were shown as mean ± SD (n = 3 biological replicates). ns = not significant, ∗∗*p* < 0.01, ∗∗∗*p* < 0.001. *B*, HL-60 cells stably infected with LV-pre-miR34a were treated with PD98059 or DMSO (control). Myeloid differentiation was assessed by following 1 μM ATRA treatment for 48 h. Unpaired *t* test was performed, data were shown as mean ± SD (n = 3 biological replicates). ∗∗∗*p* < 0.001. *C* and *D*, QRT-PCR analysis of miR34a-3p (*C*) and miR34a-5p (*D*) levels in HL-60 cells treated as in (*A*). Bar charts for individual replicate (#1, #2, #3) were shown. One-way ANOVA revealed a statistically significant difference among the four groups (*p* < 0.0001), and an unpaired *t* test was performed between two groups; the data were shown as mean ± SD (n = 3 technical replicates). ∗*p* < 0.05, ∗∗*p* < 0.01, ∗∗∗*p* < 0.001, ∗∗∗∗*p* < 0.0001. *E*, western blot analysis of ISOC1 expression in HL-60 cells treated as in (*A*). One-way ANOVA revealed a statistically significant difference among the four groups (F = 5.961, *p* < 0.05), and an unpaired *t* test was performed between two groups; the data were shown as mean ± SD (n = 3 biological replicates). ns = not significant, ∗*p* < 0.05.

PD98059 is a MEK inhibitor that inhibits MEK1 and MEK2. To investigate the effect of PD98059 on ERK signaling in the presence or absence of ATRA, as well as the effect of ATRA itself on ERK signaling, HL-60 cells were treated with either 2 μM PD98059 alone, 1 μM ATRA alone, or a combination of 2 μM PD98059 and 1 μM ATRA for 48 h. The results showed that PD98059 effectively inhibited ERK signaling irrespective of ATRA, while ATRA showed no significant effect on ERK signaling at this time point (Fig. 8*A*). We then examined the effect of ERK signaling on the pro-differentiation function of miR34a. The results showed that PD98059 markedly abolished the ability of miR34a to enhance ATRA-induced myeloid differentiation, as indicated by the reduction in CD11b^+^ cells (from 19.13 ± 0.56% to 5.44 ± 0.97%, *p* < 0.001) (Fig. 8*B*). To further investigate the effect of ERK signaling on miR34a, we examined the expression changes of miR34a isoforms in HL-60 cells treated with PD98059 in the presence or absence of ATRA. PD98059 treatment significantly reduced the levels of miR34a-3p and miR34a-5p in the absence of ATRA. However, in the presence of ATRA, PD98059 treatment led to a significant reduction in miR34a-3p but an upregulation of miR34a-5p. Notably, ATRA treatment alone decreased the levels of both miR34a-3p and miR34a-5p, indicating the negative feedback of ATRA on miR34a (Fig. 8, *C* and *D*). We further examined the effect of PD98059 on ISOC1 protein expression by western blot. The results demonstrated that PD98059 treatment significantly upregulated ISOC1 protein levels in HL-60 cells regardless of the presence of ATRA, suggesting that ERK signaling negatively regulated ISOC1 expression (Fig. 8*E*). Collectively, our findings demonstrated that inhibition of ERK signaling suppressed the miR34a-3p/ISOC1 axis, thereby attenuating ATRA-induced myeloid differentiation in APL cells.

## Discussion

In this study, analysis of paired bone marrow samples from APL patients showed that both miR34a-3p and miR34a-5p levels were significantly higher at complete remission than at initial diagnosis, suggesting a positive correlation between miR34a expression and therapeutic response to ATRA. Although miR34a alone had no effect on myeloid differentiation in APL cells, it acted as a novel regulator of ATRA efficacy. Enforced expression of miR34a significantly enhanced ATRA-induced myeloid differentiation in both HL-60 and NB4 cells at multiple time points. This pro-differentiation effect was further validated in NOG xenograft mouse models, where miR34a overexpression led to reduced spleen size and an increased proportion of human CD11b^+^ cells in the bone marrow following ATRA treatment, confirming that miR34a enhanced ATRA-induced myeloid differentiation of APL cells *in vivo*. Interestingly, despite this pro-differentiation role, both miR34a-3p and miR34a-5p levels were downregulated following ATRA treatment, indicating the existence of a potential negative feedback loop of ATRA on miR34a expression.

To elucidate how miR34a enhanced ATRA-induced differentiation, we first focused on cell cycle regulators, as the close relationship between cell differentiation and cell cycle arrest (25, 26). G1/S cycle arrest contributes to cell differentiation (25, 26), and we found that overexpression of miR34a led to G1/S cell cycle arrest in our study. The entry and progression of the cell cycle from G1 phase into S phase is regulated by CDK4/6 (27). Inhibition of CDK4/6 leads to G1/S cell cycle arrest (28) and megakaryocytic differentiation of K562 cells (16). In our study, there was no significant decrease in CDK4 in ATRA-untreated APL cells, suggesting CDK4 was not a direct target of miR34a. CDK6, a validated target of miR34a-5p in miRTarBase (MIRT001012), was found to be downregulated with and without ATRA treatment, confirming that CDK6 was a target of miR34a-5p. The downregulation of CDK6 was more significant following miR34a overexpression, suggesting the involvement of CDK6 in the promoting effect of miR34a on ATRA-induced differentiation.

However, G1/S cell cycle arrest could not fully account for the promoting effect of miR34a on ATRA-induced differentiation, as miR34a exerts opposite effects on distinct differentiation lineages within the same cellular context. For instance, miR34a inhibits osteoblast differentiation (29) but promotes osteogenic commitment in mesenchymal stem cells (30). Similarly, in hematopoietic lineages, miR34a restricts B-cell maturation by blocking the pro-B to pre-B cell transition (31), yet enhances megakaryocytic differentiation (16). Therefore, it is necessary to identify the lineage-specific targets of miR34a.

ISOC1 is a member of the hydrolases family, which can hydrolyze isochorismate into 2,3-dihydroxy-2,3-dihydrobenzoate and pyruvate (32). It is significantly upregulated in colorectal cancer (33), pancreatic cancer (34), and non-small cell lung cancer (35), where it promotes tumor progression. Additionally, ISOC1 participates in innate immunity and serves as a downstream target of miR130a and miR600 (36, 37, 38, 39). In our study, GESA revealed that ISOC1 acted as a negative regulator of hematopoietic lineage differentiation in AML patients, suggesting its role as a differentiation-specific modulator. However, the mechanism underlying ISOC1-mediated regulation of hematopoietic differentiation remains unclear and warrants further investigation. Through target prediction and subsequent experimental validation, we found ISOC1 to be a novel direct target of miR34a-3p. Functional analysis demonstrated that ISOC1 suppressed ATRA-induced differentiation, whereas miR34a promoted ATRA-induced differentiation by directly targeting ISOC1.

ERK signaling is known to promote ATRA-induced differentiation in acute promyelocytic leukemia (APL) cells (18, 19), yet the underlying mechanism remains unclear. Here, we showed that ERK signaling inhibition markedly abolished the pro-differentiation function of miR34a, which also suggested that miR34a could rescue the blocking effect of ERK signaling inhibition on ATRA-induced differentiation, revealing an upstream regulator and downstream effector relationship between ERK signaling and miR34a. The downregulation of miR34a isoforms (-3p and -5p) upon ERK inhibitor treatment further validated the regulatory relationship between ERK signaling and miR34a. However, the regulatory effect of ERK on miR34a isoforms was affected by ATRA. In the presence of ATRA, ERK inhibition predominantly suppressed miR34a-3p, whereas it led to a net increase in miR34a-5p expression, which may arise from the weakening of an ATRA-mediated negative feedback loop specifically targeting this isoform. Besides, ERK signaling inhibition upregulated ISOC1 protein expression, suggesting that ERK signaling negatively regulated ISOC1. Collectively, these findings indicated that ERK signaling promoted ATRA-induced myeloid differentiation through miR34a-3p/ISOC1 axis, and the ERK/miR34a-3p/ISOC1 axis played a critical role in regulating ATRA efficiency.

The ERK signaling pathway regulates gene expression by activating downstream transcription factors. Upon activation by extracellular signals, Ras initiates the sequential activation of Raf (MAP3K), MEK (MAP2K), and finally ERK (MAPK) *via* dual phosphorylation of its key threonine and tyrosine residues. Phosphorylated ERK then translocates to the nucleus, where it directly phosphorylates and activates the ETS family transcription factor, which recruits co-activators to initiate transcription (40, 41). Among these, ELK1 is a powerful effector of ERK signaling (42, 43). In primary human fibroblasts undergoing Raf-induced senescence, ELK1 has been identified as a transcriptional activator of miR34a, directly binding to its promoter upon ERK activation (44). Similarly, in lung cancer cells, ELK1 directly binds the miR34a promoter and upregulates its expression (45). Based on these findings and our own observations in APL cells, we propose a similar regulatory mechanism between ERK signaling and miR34a in regulating ATRA efficiency, which needs further validation.

In conclusion, our work revealed miR34a enhanced ATRA-induced myeloid differentiation by targeting CDK6 *via* miR34a-5p and ISOC1 *via* miR34a-3p, ERK signaling promoted ATRA-induced myeloid differentiation through miR34a-3p/ISOC1 axis. Targeting this regulatory axis may provide a novel combinatorial strategy to improve the therapeutic efficacy of ATRA in APL.

## Experimental procedure

### Human samples

Bone marrow samples were obtained from 5 patients with APL in Weifang People’s Hospital at two timepoints: initial diagnosis and complete remission. Bone marrow mononuclear cells were isolated by RBC lysis buffer (Solarbio) according to the manufacturer’s protocol. Informed consent was obtained from all subjects involved in the study. The study was conducted in accordance with the Declaration of Helsinki and was approved by the Medical Research Ethics Committee of Shandong Second Medical University.

### Cell cultures

The APL cell lines HL-60 and NB4 were kindly provided by Cell Bank, Chinese Academy of Sciences and Procell co., Ltd, respectively. The two lines were authenticated by STR profiling. HL-60 cells were maintained in IMDM (Gibco) supplemented with 15% fetal bovine serum (FBS). NB4 cells were cultured in RPMI 1640 supplemented with 10% FBS at 37 °C in a humidified atmosphere consisting of 5% CO_2_.

For myeloid differentiation, cells were seeded in 6-well plates at a density of 2 × 10^5^/ml, 2.5 ml per well, with individual culture medium. ATRA was obtained from Sigma-Aldrich and dissolved in DMSO. ATRA was added from a 2 mM stock in DMSO to make a final concentration of 2 μM in HL-60 cells (46) and 1 μM in NB4 cells (47), respectively.

PD98059 was purchased from MedChemExpress (MCE) and dissolved in DMSO. For experiments treated with PD98059, cells were seeded in 6-well plates at a density of 5 × 10^5^/ml 8 h prior to the first treatment with 2 μM PD98059. 16 h after the first treatment with PD98059, cells were re-cultured at a density of 2 × 10^5^/ml, and a second treatment of 2 μM PD98059 was performed. After 40 h, a third treatment of 2 μM PD98059 was performed (18). For induction of myeloid differentiation, 1 μM ATRA was added together with the second addition of PD98059. During ATRA-induced myeloid differentiation, PD98059 was added every 16 h.

### Construction of LV-pre-miR34a, LV-ISOC1, LV-ISOC1-shRNA recombinant lentiviral vectors and cell infection

LV-pre-miR34a homolog, LV-ISOC1 homolog, and LV-ISOC1-shRNA recombinant lentiviral vectors were constructed by GenePharma Co, Ltd (Shanghai, China). PCR products were cloned into a lentiviral vector using XmnI (Fermentas), NotI restriction enzymes, and T4 DNA ligase. Subsequently, lentivirus was produced from the transformation of the shuttle plasmid and packaging plasmid (pGag/Pol, pRev, pVSV-G) in HEK 293T cells. The sequences of homologs and shRNA are provided in Table 1.

**Table 1:** The sequences of homologs and shRNA in recombinant lentivirus

Homologs/shRNA	Sequences (5′-3′)
LV-Pre-miR34a	GGCCAGCUGUGAGUGUUUCUUUGGCAGUGUCUUAGCUGGUUGUUGUGAGCAAUAGUAAGGAAGCAAUCAGCAAGUAUACUGCCCUAGAAGUGCUGCACGUUGUGGGGCCC
LV-ISOC1	ATGGCGGCTGCGGAGCCGGCGGTCCTTGCGCTCCCCAACAGCGGCGCCGGGGGCGCGGGGGCGCCGTCGGGCACAGTCCCGGTGCTCTTCTGTTTCTCAGTCTTCGCGCGACCCTCGTCGGTGCCACACGGGGCGGGCTACGAGCTGCTCATCCAGAAGTTCCTCAGCCTGTACGGCGACCAGATCGACATGCACCGCAAATTCGTGGTGCAGCTGTTCGCCGAGGAGTGGGGCCAGTACGTGGACTTGCCCAAGGGCTTCGCGGTGAGCGAGCGCTGCAAGGTGCGCCTCGTGCCGTTGCAGATCCAGCTCACTACCCTGGGAAATCTTACACCTTCAAGCACTGTGTTTTTCTGCTGTGATATGCAGGAAAGGTTCAGACCAGCCATCAAGTATTTTGGGGATATTATTAGCGTGGGACAGAGATTGTTGCAAGGGGCCCGGATTTTAGGAATTCCTGTTATTGTAACAGAACAATACCCTAAAGGTCTTGGGAGCACGGTTCAAGAAATTGATTTAACAGGTGTAAAACTGGTACTTCCAAAGACCAAGTTTTCAATGGTATTACCAGAAGTAGAAGCGGCATTAGCAGAGATTCCCGGAGTCAGGAGTGTTGTATTATTTGGAGTAGAAACTCATGTGTGCATCCAACAAACTGCCCTGGAGCTAGTTGGCCGAGGAGTCGAGGTTCACATTGTTGCTGATGCCACCTCATCAAGAAGCATGATGGACAGGATGTTTGCCCTCGAGCGTCTCGCTCGAACCGGGATCATAGTGACCACGAGTGAGGCTGTTCTGCTTCAGCTGGTAGCTGATAAGGACCATCCAAAATTCAAGGAAATTCAGAATCTAATTAAGGCGAGTGCTCCAGAGTCGGGTCTGCTTTCCAAAGTATAG
LV-ISOC1-shRNA	ATTTGAGTACCAGCATTTA
LV-scramble	TTCTCCGAACGTGTCACGT

HL-60 and NB4 cells were grown in 24-well plates containing a density of 2 × 10^5^/ml, 500 μl per well in fetal bovine serum (FBS)-free culture medium. Cells were first centrifuged at 1000 rpm for 5 min, then infected with lentivirus at an MOI of 50 in the presence of polybrene (GenePharma). Stable clones were screened and amplified in medium containing puromycin.

### Flow cytometric analysis

Cells were stained with FITC/APC-conjugated mouse anti–human CD11b antibody (eBioscience) or PE -conjugated mouse anti–human CD45 antibody (eBioscience). Corresponding isotype control antibodies (eBioscience) were used to set the gating threshold. Stained cells were measured using BD FACSCalibur (BD Bioscience). A minimum of 10,000 events was collected for each sample. The data were analyzed using FlowJo software.

Cell cycle was also assessed by flow cytometric analysis. 2 × 10^6^ cells were fixed with 70% pre-cooled ethanol and incubated at 4 °C for 24 h. Then, cells were re-suspended in 50 μl PBS containing 500 μl propidium iodide solution (Beyotime, Shanghai, China) and incubated for 30 min at room temperature in the dark. The DNA content was evaluated using the FACSCalibur flow cytometry system.

### RNA isolation and QRT-PCR

Total cellular RNA, including small RNAs, was isolated using Trizol (Thermofisher Scientific). For mRNA detection, reverse transcription was performed using PrimeScript RT Master Mix (Takara, Japan), and the QRT-PCR was carried out using One Step TB Green PrimeScript RT-PCR Kit (Takara, Japan). GAPDH was used as an endogenous control. For miRNA detection, reverse transcription was performed using Mir-X miRNA First-strand Synthesis kit (ACCURATE BIOTECHNOLOGY Co, Ltd), and the QRT-PCR was carried out using Mir-X miRNA QRT-PCR TB Green Kit (ACCURATE BIOTECHNOLOGY Co, Ltd). U6 was used as an endogenous control. Relative expression of predicted mRNAs or miRNAs was shown with the comparative Ct method (2^−ΔΔCt^). mRQ 3′ primer was provided by the miRNA first strand cDNA synthesis kit. The 5′ primers of individual miRNA and the primers of mRNA were listed in Table 2.

**Table 2:** The sequences of primers in QRT-PCR analysis

Primer	Sense(5′-3′)	Antisense(5′-3′)
miR34a-3p	CAATCACCATCTATACTGCCCT	
miR34a-5p	TGGCAGTGTCTTAGCTGGTTGT	
U6	GCGCGTCGTGAAGCGTTC	GTGCAGGGTCCGAGGT
*ISOC1*	AGCGGCATTAGCAGAGATTCC	TGAGGTGGCATCAGCAACAAT
*GAPDH*	ACAACAGCCTCAAGATCATCAG	TCTTCTGGGTGGCAGTGATG

### Western blot analysis

Total proteins were separated by SDS-PAGE gel electrophoresis followed by electroblotting onto PVDF membranes. After blocking, the membrane was incubated with specific primary antibodies against CDK1 (PB9533, Boster), CDK2 (BM3926, Boster) CDK4 (BM4672, Boster, Wuhan, China), CDK6 (BM4671, Boster), Cyclin D1 (BM4272, Boster), Cyclin E1 (A00543–2, Boster), ISOC1 (PA5-98681, Invitrogen), p-ERK1/2 (4370, CST, USA), ERK1/2 (4695T, CST), β-actin (80,624, ZenBio), GAPDH (380,626, ZenBio), and then incubated with the corresponding HRP-labelled secondary antibodies. Specificity for CDK1, CDK2, CDK4, CDK6, Cyclin D1, Cyclin E1, p-ERK1/2, ERK1/2, β-actin, and GAPDH antibodies was confirmed by the validation data supplied by the manufacturers, and specificity for ISOC1 antibody was validated by the *ISOC1* knockdown experiment. Target proteins were detected using Amersham ImageQuant 800 Western blot imaging systems (Cytiva). Three independent experiments were performed.

### Datasets and gene set enrichment analysis (GSEA)

The RNA sequencing (RNA-seq) data of 179 AML samples in the TCGA database were downloaded from the UCSC Xena database (https://xena.ucsc.edu/). AML samples were divided into high-*ISOC1* and low-*ISOC1* groups. High-*ISOC1* group was defined as the samples with *ISOC1* expression higher than the median, while low-*ISOC1* group was defined as the samples with *ISOC1* expression lower than the median. GSEA was used to identify enriched biological pathways associated with *ISOC1* in AML using Sangerbox tools (http://vip.sangerbox.com/). |NES|>1, *p*-value < 0.05 and FDR < 0.25 were considered statistically significant.

### Dual-luciferase reporter assay

The target genes of miR34a-3p were predicted by the miRanda database and validated by dual-luciferase reporter assay. miR34a-3p and normal control (NC) mimics were synthesized. GP-mirGLO plasmid (GenePharma), consisting of firefly luciferase gene, Renilla luciferase gene, and multiple cloning site region, was used to construct miR34a-3p inhibitor sponge, wild-type *ISOC1* (*ISOC1*-WT), and mutant *ISOC1* (*ISOC1*-MUT) plasmids. 293T cells were divided into three groups: Vector, *ISOC1*-WT, and *ISOC1*-MUT. For each group, cells were transiently infected with 50 pmol mimics and 1 μg plasmid by GP-transfect-Mate (GenePharma) according to the manufacturer’s protocols. Firefly luciferase and Renilla luciferase activities were determined 48 h after transfection using a dual-luciferase assay kit (GenePharma). Relative firefly luciferase activities were achieved by normalization of firefly luciferase activities to Renilla luciferase activities.

### NOG xenograft mouse model

6 male NOD.Cg-Prkdc^scid^Il2rg^tm1Sug^/JicCrl (NOG) mice (6 weeks old, body weight 16–18*g*) were bought from Vitalstar Biotechnology Co, Ltd (Beijing, China) and divided equally into two groups: LV-vector and LV-pre-miR34a. Two days prior to tail vein injection, each mouse received an intraperitoneal injection of busulfan (20 mg/kg) to facilitate cell engraftment (23). Subsequently, 2 × 10^6^ HL-60 cells stably infected with LV-vector-Puro or LV-pre-miR34a-Puro recombinant lentiviral vector were intravenously injected *via* the tail vein. Two weeks after transplantation, peripheral blood human CD45 (hCD45) levels were analyzed by flow cytometry. Once hCD45^+^ cells exceeded 1%, mice were treated with ATRA (5 mg/kg/d) by gavage for 10 days to induce myeloid differentiation. Mice were euthanized posttreatment, and bone marrow, liver, and spleen were harvested. Ethical approval for this study was granted by the Animal Research Ethics Committee of Shandong Second Medical University.

### Statistical analysis

Data were analyzed using GraphPad Prism software (version 10.6) and were presented as mean ± standard deviation (SD). For comparisons between groups, one-way analysis of variance (ANOVA) was performed, followed by an unpaired *t* test. Alternatively, an unpaired *t* test was used to compare two groups. A *p* value of less than 0.05 was considered statistically significant ∗ *p* < 0.05, ∗∗*p* < 0.01, ∗∗∗*p* < 0.001.

### Data availability

The data that support the findings of this study are available upon reasonable request from the corresponding author.

## Conflict of interest

The authors declare that they have no conflicts of interest with the contents of this article.
